# Maternal and Infant Characteristics and Pumping Profiles of Women That Predominantly Pump Milk for Their Infants

**DOI:** 10.3390/nu17020366

**Published:** 2025-01-20

**Authors:** Zoya Gridneva, Ashleigh H. Warden, Jacki L. McEachran, Sharon L. Perrella, Ching Tat Lai, Donna T. Geddes

**Affiliations:** 1 School of Molecular Sciences, The University of Western Australia, Crawley, WA 6009, Australia; ashleigh.warden@uwa.edu.au (A.H.W.); jacki.mceachran@uwa.edu.au (J.L.M.); sharon.perrella@uwa.edu.au (S.L.P.); ching-tat.lai@uwa.edu.au (C.T.L.); donna.geddes@uwa.edu.au (D.T.G.); 2 UWA Centre for Human Lactation Research and Translation, Crawley, WA 6009, Australia; 3 ABREAST Network, Perth, WA 6000, Australia

**Keywords:** lactation, breastfeeding, human milk, breast pumping, breast expression, electric breast pump, predominant pumping, exclusive pumping, milk production, milk intake

## Abstract

Background: Whilst it is inconvenient and time-intensive, predominantly (PP) and exclusively pumping (EP) mothers rely on breast expression to provide milk for their infants and to ensure continued milk supply, yet these populations are poorly understood. Methods: We assessed and characterised Western Australian PP mothers (*n* = 93) regarding 24 h milk production (MP) and infant milk intake and demographics, perinatal complications and breastfeeding difficulties, the frequencies of which were compared with published general population frequencies. Pumping efficacy and milk flow parameters during a pumping session in PP mothers (*n* = 32) were compared with those that pump occasionally (reference group, *n* = 60). Results: PP mothers had a higher frequency of pregnancy complications and breastfeeding difficulties than the general population. Exclusive pumping did not impact the 24 h MP and the amount of milk available to the infant. PP mothers had more milk ejections, longer active flow duration and lower milk removal efficacy ratios; however, responsiveness to pump (time to first milk ejection), total flow duration, time to stop pumping, percentage of available milk removed and comfort parameters were not different to the reference group. Conclusions: Despite the reported challenges, when an effective pump is used, predominant or exclusive pumping does not negatively affect the 24 h MP and the amount of milk available to the infant. Increasing awareness of the impacts of perinatal and breastfeeding complications on women may assist health professionals in early identification of mothers at high risk for early cessation of breastfeeding, escalating early postpartum intervention and targeted breastfeeding support.

## 1. Introduction

Breastfeeding is a major nutrition and public health focus; it provides multiple dose-related health benefits to breastfeeding mothers and infants [1], yet the information on human milk feeding in vulnerable infants and lactation support and care is scarce [2]. Breast pumps may be used to overcome challenges such as attachment difficulties and support milk production (MP) at various stages of lactation [3,4,5,6], and evidence shows that lactating mothers in developed settings often use a breast pump, particularly during the early months of lactation, with some predominantly or exclusively pumping for their infants [7]. While there are multiple reasons why mothers predominantly or exclusively pump, the most common reasons are infant latching difficulties and breast refusal, resulting in inadequate milk transfer when breastfeeding [8]. Other reasons include congenital conditions, preterm/sick infants that are too weak/unwell to feed directly at the breast and maternal preference. With the use of breast pumps is generally increasing [9], predominantly pumping (PP) as well as exclusively pumping (EP) mothers [8,10] rely on expressing to provide milk for their infants and to ensure continued milk supply.

Whilst predominant or exclusive pumping can increase the proportion of infants that are fed any breast milk and thus reduce the risks of commercial milk formula feeding, little is known about these populations. A recent retrospective survey of a small group (*n* = 33) of EP women has indicated that those that exclusively pump are a diverse group that may differ demographically from women that breastfeed or pump occasionally and that they may have a shorter lactation duration [10]. Additionally, a scoping review concluded that EP mothers are an underserved population, finding no publications on formal and informal community support for EP mothers [11]. Furthermore, a recent systematic review that aimed at examining the weight trajectories of breastfed and bottle-fed infants found that only 31 infants out of 5152 were fed predominantly or exclusively expressed breast milk [12]. This emphasises the need to expand our understanding of these dyads and their needs, as these populations are growing, and with limited healthcare provider knowledge, women typically source information on predominant and exclusive pumping from social media [8,13,14], whilst antenatal breastfeeding education classes rarely include information on exclusive pumping [15,16].

This pilot cross-sectional study aimed to assess and characterise PP mothers from high income settings, both with normal and low milk supply, in relation to demographics and 24 h MP. We also aimed to assess and compare the pumping efficacy and milk flow parameters of PP mothers and those who pump occasionally.

## 2. Materials and Methods

### 2.1. Study Design and Participants

**Figure 1:**
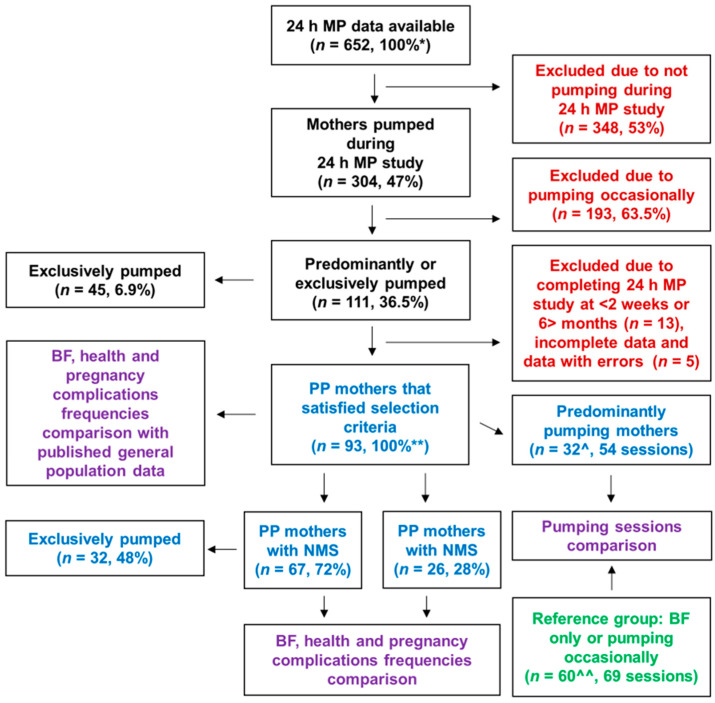
Study flow and analysis diagram. BF, breastfeeding; LMS, low milk supply; MP, milk production; NMS, normal milk supply; PP, predominantly pumping. * Indicates 100% of all available participants’ data; ** indicates 100% of selected participants. ^ Includes 24 selected PP mothers and 8 PP mothers without 24 h MP data; ^^ includes 15 mothers from the excluded participants that were breastfeeding only or pumping occasionally. Black font colour indicates the entire REDCap sample; red font colour—excluded participants; blue font colour—PP mothers; green font colour—reference group participants; purple font colour—comparisons between the groups.

This is a retrospective analysis of cross-sectional 24 h MP, infant feeding and breast expression data that were collected primarily between 2020 and 2023 (2013–2024) as a part of various breastfeeding studies conducted in the group. The University of Western Australia’s Research Electronic Data Capture (REDCap) records (*n* = 652) were searched for participants (PP mothers) that expressed more than 50% of their total 24 h milk volume removed during a single 24 h milk profile study (Figure 1). Participants lived in the Perth metropolitan area, Western Australia, and included those with normal milk supply (NMS group, ≥600 mL/24 h) as well as women with low milk supply (LMS group, <600 mL/24 h) [17,18]. The selection criteria included English speaking, non-smoking mothers of singleton infants between 2 weeks and 6 months postpartum, as achievement of secretory activation and coming to volume by 2 weeks postpartum (≥500 mL/24 h) is considered predictive of normal milk production even in mothers of vulnerable infants [19], and infant milk intake between 1 and 6 months of age is considered stable [18,20].

The selected PP mothers’ maternal health conditions and perinatal complications and breastfeeding difficulties frequencies were compared between NMS and LMS groups and further with the published frequencies of these conditions in the general population. The comparison to the general population instead of the fully breastfeeding and/or occasionally pumping participants that were excluded from the study was chosen, as our sampling method may be biased in that participants with breastfeeding challenges may have sought research participation to address these challenges.

Additionally, the PP mothers’ milk removal during pumping session data were compared with the data of a reference group that fully breastfed or pumped occasionally. Data for the reference group were collected earlier (2017–2019), prior to using the REDCap application; some of these participants (*n* = 15) were added to REDCap (for ongoing studies) and were part of the excluded participants (Figure 1).

The studies were conducted in accordance with the Declaration of Helsinki and were approved by The University of Western Australia Human Research Ethics Committee (2019/RA/4/20/6134 and 2019/RA/4/20/6407). Informed written consent was obtained from all participants.

### 2.2. Twenty-Four-Hour Milk Profile Data Collection

Twenty-four-hour MP measurements were conducted using the test-weighing method [18,20]. Briefly, weights of milk collection bottles before and after any manual or pump breast expressions as well as pre- and post-feed weights of infants were recorded using electronic scales (±2.0 g; Electronic Baby Weigh Scale, Medela Inc., McHenry, IL, USA) for all feeds/expressions in one 24 h period plus one feed/expression. All expressed milk or feed weights were recorded and reported in grams (human milk density = 1.03 g/mL) [21]. Twenty-four-hour MP was calculated using the formula below, where *v_i_* is the volume of each breastfeed/expression, *N* is the total number of breastfeeds and expressions, and *T* is the elapsed time from the end of the first breastfeed/expression until the end of the last breastfeed/expression.

$$MP=\sum_{i=2}^{N}v_{i}\frac{24}{T}$$

The PP mothers were classified into two groups: NMS and LMS; a small group of mothers from the NMS group that exclusively pumped (EP, *n* = 32) was also characterised. Milk removal frequency (combined breastfeeding and breast expression frequencies) was recorded from the 24 h MP data. Mothers completed questionnaires regarding demographic, obstetric and infant details, including maternal age, parity, birth gestation, birth mode, perinatal and health complications and breastfeeding difficulties.

### 2.3. Milk Removal Data Collection

A subgroup of PP mothers (*n* = 32) also participated in one to four pumping sessions (*n* = 54) where one breast was pumped using a hospital-grade electric breast pump (Medela AG, Baar, Switzerland) and a fitted breast shield (to provide a breast shield diameter ~4 mm wider than the nipple base diameter). Participants attended the research room at The University of Western Australia for the pumping session. The pump vacuum was set to the participants’ maximum comfort level and adjusted when necessary for the participants’ comfort [22]. Milk output and removal parameters were measured together with participant comfort ratings at each session. Comfort levels were rated at the start and at the end of the pumping session with mothers using a scale from 1 to 5 (1—very comfortable; 2—comfortable; 3—neither comfortable nor uncomfortable; 4—uncomfortable; 5—very uncomfortable). Pumping sessions were 15 min in duration, commencing at the start of the first milk ejection.

**Figure 2:**
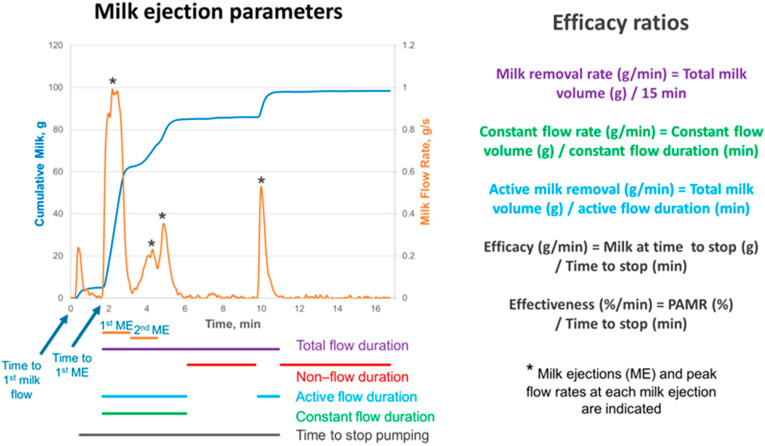
Milk ejection and milk flow parameters, including flow durations and efficacy ratios. The graph presents an example milk flow pattern with four milk ejections during 15 min breast expression. The blue line indicates the cumulative weight of milk (g) and the orange line indicates the milk flow rate (g/s). The horizontal lines beneath the graph represent flow durations during expression with corresponding colour-coordinated efficacy ratios (with exception of non-flow duration) on the right of the graph. Time to stop pumping (min)—time after which 0–<10% of milk is removed. ME, milk ejection; PAMR, percentage of available milk removed.

A continuous weighing balance was used to determine changes in milk flow rate and time to first milk flow and first milk ejection (first jets of milk from the nipple, min) [23]. The recording of the cumulative weight (g) of the milk and the milk flow rate (g/s) allowed for the calculation of milk ejection and milk removal parameters, including flow durations and efficacy ratios (Figure 2).

Nipple temperature was determined pre- and post-expression with the FLIR T650sc thermal camera with a thermal sensitivity of <20 mK @ 30 °C (±0.02 °C; Flir Systems Inc., Wilsonville, OR, USA) from the acquired images using the provided FLIR software (FLIR Research IR, version 4.30.1.70, Wilsonville, OR, USA) as described previously [24]. The nipple was gently dried to remove any remaining milk prior to acquiring the images. All equipment in contact with breast milk was sterilised before use and expressed milk was returned to the participant.

During the 24 h MP measurement, PP mothers that participated in pumping sessions also collected small (<2.0 mL) milk samples pre- and post-expressions and/or breastfeeds by manual expression into 5 mL polypropylene tubes (P5016SL, Techno Plas Pty Ltd., St. Marys, SA, Australia). Samples were stored in the participant’s home freezer until they were transported to the laboratory, where fat concentration was analysed using the creamatocrit method [25]. Using the pre- and post-expression fat concentrations at the pumping session and participants’ 24 h MP data, the degree of fullness of the breast (DOF) pre- and post-expression as well as the percentage of available milk removed (PAMR) were calculated based on the method described previously [22,26]. The PAMR results were not capped where the calculated values exceeded 100%.

Measures of efficacy of milk removal and comfort during pumping were compared between the PP subgroup and a reference group (*n* = 60) that fully breastfed or pumped occasionally and also completed a pumping session with the standard Symphony pattern (*n* = 69 sessions) and 24 h MP (Figure 1).

### 2.4. Statistical Analyses

Descriptive statistics are presented as mean and standard deviation for continuous variables and frequencies/counts and percentages for categorical variables. Statistical analysis used unpaired Students’ *t*-tests to compare continuous variables such as 24 h MP measurements between the groups, milk flow parameters between PP mothers and the reference group, and Chi-square or Fisher’s exact tests for binary variables in terms of infant feeding practises, demographics, maternal health conditions and perinatal complications, and breastfeeding difficulties between the NMS and LMS groups. The Chi-square test was also used for comparing the PP group’s maternal health and perinatal complications and the frequencies of breastfeeding difficulties with published frequency data from general populations reported in Australia [27,28,29,30,31,32,33]), Brazil [34], Italy [35], Russian Federation [36] and USA [37,38]. Missing data were addressed using available case analysis (pairwise deletion) to preserve the sample sizes and to have a higher efficiency and avoid bias and loss of precision [39]. The significance level in this investigative study was set at *p* < 0.05.

## 3. Results

### 3.1. Participants’ Demographic, Perinatal and Breastfeeding Characteristics

From 652 REDCap records, 304 mothers (47%) expressed during a 24 h MP study; 63.5% of them (*n* = 193) expressed occasionally (expressed in total less milk than breastfed), 36.5% (*n* = 111) predominantly pumped and 6.9% (*n* = 45) exclusively pumped. Of 93 PP mothers that satisfied the selection criteria, 67 (72%) had NMS and 26 (28%) had LMS. Forty-one mothers (44.1%) exclusively expressed at the time of the study (NMS: *n* = 32, 47.8%; LMS: *n* = 9, 34.6%). The LMS group performed the 24 h MP study earlier than the NMS group (*p* = 0.008; Table 1). Most participants were primiparous (*n* = 70/93, 75%) and gave birth at term (*n* = 83/91, 91%), with 53% PP mothers birthing by caesarean section.

Fifty-eight percent of PP mothers reported perinatal complications, such as gestational diabetes mellitus (GDM; 28%) and gestational hypertension (8%), and at least one maternal health condition (55%) including anxiety/depression (28%) and polycystic ovary syndrome (19%) (Table 2). All these conditions, except for polycystic ovary syndrome, were more frequently reported by PP mothers compared to the general population (Table 3). Infant health conditions were reported in 21.5%.

The most frequently reported breastfeeding/pumping concerns apart from perceived low milk supply were attachment difficulties (49%) and nipple pain (41%), followed by nipple shield use (31.5%), blocked ducts (15%) and mastitis (8%) (Table 2). Perceived low milk supply was reported by 40% of mothers, with 20% in the EP group (5/25, average MP 781 ± 105 g, range: 659–898 g), 24% in the NMS group (13/54, average MP 739 ± 88 g, range: 628–898 g) and 84% in the LMS group (*p* < 0.001). All LMS group mothers reported breastfeeding difficulties, compared to 78% in the NMS group (*p* = 0.029). Nine percent of NMS mothers reported oversupply. Attachment difficulties and nipple shield use were more frequently reported by PP mothers compared to the general population (Table 3).

### 3.2. Twenty-Four-Hour Pumping and Breastfeeding Characteristics

**Figure 3:**
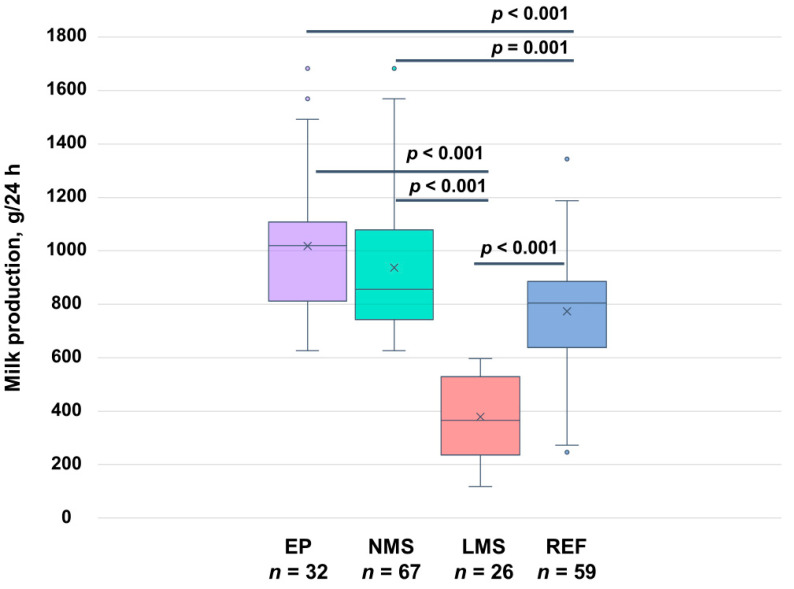
Twenty-four-hour milk production by group. EP, exclusively pumping mothers; LMS, low milk supply; NMS, normal milk supply; REF, reference group of breastfeeding and occasionally pumping mothers. *p*-values indicate differences between the groups.

The 24 h MP in NMS group was 937 ± 263 g (range: 626–1682 g), which was significantly higher than in the LMS group (379 ± 149 g; range: 118–597 g; *p* < 0.001) (Table 4) and in the reference group (*n* = 59; 773 ± 233 g; range: 246–1344 g; *p* < 0.001) (Figure 3). Fifty-two percent of the NMS group (35/67) and 65% of the LMS group (17/26) were breastfeeding, with no difference in 24 h breastfeeding frequency, the average or total 24 h milk volume breastfed between the groups. However, within the breastfeeding subgroups, the average breastfed amount was higher in the NMS than in the LMS group (38.4 ± 31.4 g vs. 21.0 ± 16.1 g, respectively, *p* = 0.036).

Most mothers were performing simultaneous pumping (NMS: 88%; LMS: 92%), i.e., both breasts at the same time and pumping at night (NMS: 83%; LMS: 81%). Overall, the average (*p* = 0.002) and total amount (*p* < 0.001) of expressed breast milk (EBM) and breast milk (*p* < 0.001) fed to the infant in 24 h was significantly higher in the NMS group and the amount of formula fed was lower (*p* < 0.001).

Twenty-two percent (15/67) of mothers from the NMS and 85% from the LMS (22/26) group supplemented with formula. Within the subgroups supplementing with formula, the 24 h formula feed frequency and the total 24 h formula amounts fed were lower in the NMS than in the LMS group (formula feeds: 2.4 ± 1.9 g vs. 4.9 ± 2.4, respectively, *p* = 0.001; total 24 h formula amount: 205 ± 130 g vs. 391 ± 197 g, respectively, *p* = 0.002), whilst the average formula feed amount was not different (96 ± 42 g vs. 87 ± 48 g, respectively, *p* = 0.54).

**Figure 4:**
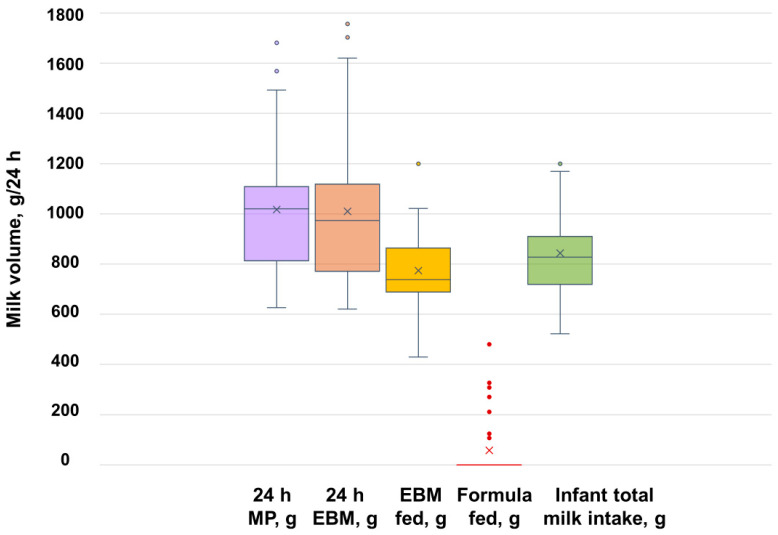
Twenty-four-hour milk production and milk amounts fed to the infant in the group of exclusively pumping mothers (*n* = 32). EBM, expressed breast milk; MP, milk production.

In EP mothers (*n* = 32), the 24 h MP was 1018 ± 274 g (range: 626–1682 g) and was significantly higher than in the reference group (*n* = 60; 792 ± 219 g; range: 278–1274 g; *p* < 0.001) (Table 4, Figure 3). The infant intake of EBM was 773 ± 184 g (range: 430–1200 g) (Table 4, Figure 4) and the majority of EP mothers’ infants (23/26, 88.5%) consumed more than 600 g of EBM. Only seven participants fed formula during 24 h MP (261 ± 129 g; range: 106–480 g) (Figure 4).

### 3.3. Milk Removal Parameters During Pumping Session

In the subgroup of PP mothers that participated in pumping sessions (*n* = 32, 54 sessions), most of the milk removal parameters during pumping sessions were similar to that of the reference group (*n* = 60, 69 sessions) (Table 5). The 24 h MP was higher in PP mothers (962 ± 338 g; range: 274–1682 g) compared to the reference group (773 ± 233 g; range: 246–1344 g) (*p* = 0.004). PP mothers completed pumping sessions at the earlier time postpartum; there was no difference in frequency of LMS between the groups (Table 5).

In PP mothers, the number of milk ejections was higher, whilst peak flow rates and milk removed during both, first and second milk ejections were lower (Table 6). In PP mothers, active flow duration was longer and non-flow duration was shorter, whilst total and constant flow durations and time to stop pumping were not different.

In PP mothers, the milk removal and constant flow rates and efficacy ratio were similar to the reference group mothers; however, the active milk removal ratio and effectiveness ratio as well as milk removed after time to stop pumping were lower in PP mothers (Table 7). In PP mothers, nipple temperature changes (°C) from pre- to post-expression and initial and final comfort were similar. However, PP mothers selected weaker expression vacuum.

## 4. Discussion

In this study, PP mothers had a higher prevalence of pregnancy complications, caesarean birth, mental health challenges and breastfeeding issues than the general population. When compared to women that occasionally pumped, PP mothers had more milk ejections, lower efficacy ratios and longer active flow durations (likely related to their lower expression vacuums). However, the responsiveness to pump (time to first milk flow and to first milk ejection) as well as total flow duration, time to stop pumping, PAMR and comfort parameters were similar to the reference group. Importantly, exclusive pumping did not appear to impact 24 h MP and the amount of milk that could be offered to the infant. All EP mothers in our study were able to sustain lactation above 600 g/24 h (100%), and the majority (86%) provided only breast milk to their infants.

### 4.1. Breastfeeding Difficulties

Exclusive breastfeeding for 6 months with continued breastfeeding for up to two years and beyond along with complementary foods is recommended as it is estimated to prevent hundreds of thousands of deaths annually [40]. However, whilst most mothers in high-income countries, where breast pumps are used more frequently [41,42], initiate breastfeeding, at 6 months of age only 55% of infants are fed any breast milk [43], and the global prevalence of breastfeeding at 12 months in these countries is less than 20% [40]. According to a large recent Italian study (*n* = 552), factors influencing breastfeeding duration and cessation are breastfeeding difficulties, including perceived insufficient milk supply, nipple pain, fatigue and attachment difficulties, which were reported by over 70% of mothers [35]. The majority of the mothers (63%) experienced these difficulties within the first month postpartum, and by the third month, only 10% of mothers were experiencing breastfeeding difficulties, whereas breastfeeding difficulties were 8 times more frequent at the corresponding times postpartum in our PP participants.

PP mothers in our study were twice as likely to report attachment difficulties and nipple shield use than in the general population (Table 2). Notably, nipple shield use was prevalent beyond 2.5 months postpartum, indicating ongoing attachment difficulties. As attachment difficulties are associated with a higher risk of non-exclusive breastfeeding [35], it is not surprising that the PP mothers were expressing their milk to establish, build and maintain milk supply and to provide their milk for infant feeding in the face of difficult breastfeeding and subsequently reduced milk transfer with breastfeeding.

The perception of low milk supply is also consistently one of the major reasons reported to cease exclusive breastfeeding [35,44]. In our study, the overall prevalence of perceived insufficient milk supply was 40%, which is congruent with Australian and international rates of 35—50% [36,44,45,46]. Whilst studies of perceived low milk supply rarely measure actual infant milk intake to confirm maternal perception, the majority of our mothers with measured LMS (84%) were aware their milk supply was not adequate for their infant. This finding is also in line with 30%—80% of mothers citing perceived insufficient milk supply as the reason for commercial milk formula supplementation [44] and ceasing breastfeeding [46]. This highlights the importance of conducting a systematic evaluation of women reporting insufficient MP to identify known extrinsic and intrinsic causes of LMS. These may include infrequent or inefficient milk removal due to infant medical conditions that affect an infant’s ability to breastfeed effectively, such as ankyloglossia, cleft palate or neurological issues, as well as maternal conditions, such as breast hypoplasia, breast or nipple surgery, medical conditions and related medications, and obesity and associated endocrine disruption [47,48]. Interestingly, approximately a quarter of mothers with NMS, including 20% (5/25) of EP mothers, who were producing on average 739 and 781 g of milk a day, respectively, which are close to the average infant milk intake at a similar age [18], were still concerned about their milk supply. Timely evaluation of infant growth and education on infant behaviour may address misperceptions/expectations of women on how much EBM their infant needs and if their milk supply is adequate rather than relying on social media for guidance [49,50].

### 4.2. Pregnancy Complications and Mental Health

Further to the high rates of reported breastfeeding difficulties our PP mothers also reported 11% higher rates of pregnancy complications than the general population, such as GDM, gestational hypertension as well as caesarean birth and anxiety and depression (10%, 5%, 14%, and 12% higher than the general population, respectively; Table 1 and Table 2). Given the PP women’s high rates of health conditions and breastfeeding challenges, the commitment to predominant expression to produce enough milk for their infant instead of weaning is evident.

GDM rates in Australia increased from 13% in 2019 [27] to 17.9% in 2022 [29]. GDM may be associated with a higher risk of LMS [51], with only one-third of Thai and UK women with GDM breastfeeding their infants until six months of age [52,53]. Interestingly, 40% of PP mothers reported LMS, indicating that GDM per se may have an impact on the ability to produce milk. Further, our participants differed to a study of EP mothers that had a lower average socioeconomic status, earlier formula introduction and shorter lactation duration [10], in that 74% of women were highly educated and, therefore, more likely continued to pump to improve milk supply by frequent, regular milk removal [54,55].

Previous research has highlighted that undergoing a caesarean section is associated with delayed breastfeeding and the initiation of lactation and subsequently earlier cessation and lower rates of exclusive and any breastfeeding [56,57,58]. In our study, mothers had a higher rate of caesarean birth, which likely contributed to their decision to pump. Further, despite NMS mothers having the highest elective caesarean birth rate (35.4%), the majority had no issues with milk supply, suggesting that multiple factors other than caesarean section may be impacting milk synthesis, which could be identified during antenatal screening [58,59].

Stress experienced by dyads during labour and delivery correlates with a delay in breast fullness and lower milk volume in early lactation [60]. Breastfeeding difficulties have been associated with a greater risk of developing depressive symptoms in the postnatal period [61], and the mental health issues, such as anxiety and depression, in our PP mothers may be reflective of the challenges they encounter during pregnancy and lactation period. However, it is not clear if these were pre-existing mental health conditions or whether these occurred after birth. Thus, it is important to provide these mothers with adequate antenatal and postnatal breastfeeding education and support to ensure expectations and strategies to impact both mental wellbeing and breastfeeding success [35,62].

### 4.3. Pumping vs. Breastfeeding—24 h Milk Production and Infant Milk Intake

Pumping is commonly believed to result in less effective milk removal than that achieved by the breastfed infant. However, considering the degree of breast fullness, the comparison between an infant feeding to appetite and an electric pump is incongruous. The milk ejection reflex is a robust physiological process [63], and the pattern of milk ejections has been shown to be similar within women during breastfeeding and pumping [64]. This indicates that if an effective and comfortable vacuum is used during pumping, appropriate periodic milk removal could sustain the lactation. Further, the infant removes on average 67% of the available milk during a breastfeed, only ‘emptying’ the breast once a day [18], whilst pumping with the Medela 2-phase expression pattern has shown to remove up to 75% of milk [65,66,67]. Indeed, MP was increased by 15 to 40% when a breast pump was used to remove additional milk after breastfeeds [68,69]. Interestingly, mothers who occasionally expressed breast milk were more likely to continue breastfeeding past six months than those who had never expressed [70], suggesting that pumping can provide maternal benefits such as time away from the infant for self-care that may contribute to continued lactation [42].

When comparing the milk flow parameters during pumping sessions, it was further established that the responsiveness to pump (time to first milk flow and first milk ejection) as well as total flow duration, PAMR, time to stop pumping and comfort parameters were not compromised in PP mothers compared with the reference group. PP mothers may have more milk ejections, longer active flow durations and lower efficacy ratios, all of which could be due to choosing weaker expression vacuums (maximum comfortable vacuum).

In our study, 71% of PP mothers, including all EP mothers, were able to sustain MP above 600 g in 24 h at 2.5 months postpartum, and most of the EP mothers (86%) provided only breast milk to their infants at 3.5 months postpartum. Nevertheless, 29% of PP mothers had LMS at the time of the study. As mothers with both LMS and NMS had the same rates of GDM and same 24 h breastfeeding and expressing frequency, LMS may have non-modifiable causes, such as genetics, for which frequent milk removal is not going to solve the problem [71].

### 4.4. Pumping vs. Breastfeeding—More Information and Support Is Needed

There is a dearth of research on predominantly and exclusively pumping families, and on methods of milk expression in lactating women in general [72]. Our study reveals that when health issues arise and direct breastfeeding is unsuccessful, modern mothers adapt and exclusively or predominantly pump to ensure their infants receive the benefits of breast milk, and that this can be successfully maintained during established lactation. Women that have access to exclusive pumping information prior to giving birth report they feel more knowledgeable and confident while exclusively pumping [15,73]; thus there is a need for a timely, accurate and helpful advice from health professionals when pregnancy complications and breastfeeding challenges arise.

The strength of our study is that the 24 h milk profile was assessed using a validated reference method [74], enabling assessment of MP and infant milk intake in this population, rather than the duration of exclusive or any breastfeeding. Our study also has several limitations; it is a small retrospective pilot cross-sectional study of mainly Caucasian, highly educated and motivated participants from a high income setting. Further, there is also a lack of current reference data for the general population for perinatal complications and breastfeeding difficulties, and our sampling method may be biased as participants with breastfeeding challenges may have sought research participation to address these challenges. Additionally, whilst using one hospital-grade pump is a strength of pumping dynamics sub-study design, the findings may be relevant only to this pump type. There is a possibility of differences in individual’s responses to different pumps, both positive and negative. Future studies could confirm or dismiss this limitation by testing the response to different pumps and should consider a randomised design accounting for the type of pump the participant routinely uses. As this was a retrospective study with the data collected over considerable time, we could not establish in detail the reasons for predominant or exclusive pumping and maternal perceptions; mixed-method approaches should be considered for future research.

## 5. Conclusions

This study found that mothers from high income settings that predominantly pump at home have a higher frequency of pregnancy complications, mental health and breastfeeding difficulties than in the general population. Despite these challenges, most predominant or exclusive pumping women were able to produce an adequate 24 h milk volume that could provide for all the infant’s milk requirements. Increasing awareness of the modifiable and non-modifiable barriers experienced by mothers during the perinatal period and breastfeeding may assist health professionals in the early identification of mothers at high risk for early cessation of breastfeeding and the implementation of early intervention and targeted breastfeeding support.

## Figures and Tables

**Table 1:** Participants’ demographic characteristics.

Maternal and Infant Characteristcs	PP (*n* = 93)	EP (*n* = 32)	NMS (*n* = 67)	LMS (*n* = 26)	*p*-Value ^2^
Maternal age at 24 h MP (years)	33.9 ± 4.3 ^1^	33.9 ± 4.9	34.0 ± 4.4	33.4 ± 4.2	0.56
Parity (primiparous)	70 (75.3)	23 (71.9)	49 (73.1)	21 (80.8)	0.59
Body mass index (kg/m^2^)	28.5 ± 6.9 ^3^	28.7 ± 4.7 ^4^	28.4 ± 7.1 ^5^	28.7 ± 6.6 ^6^	0.88
24 h MP time (months postpartum)	2.5 ± 1.6	3.4 ± 1.6	2.7 ± 1.6	1.8 ± 1.4	**0.008**
Highest maternal education level	*n* = 93	*n* = 32	*n* = 67	*n* = 26	
High school	7 (7.5)	3 (9.4)	7 (10.5)	0 (0.0)	0.18
Certificate/apprenticeship	17 (18.3)	4 (12.5)	10 (14.9)	7 (26.9)	0.23
Tertiary degree	69 (74.2)	25 (78.1)	50 (74.6)	19 (73.1)	1.00
Race	*n* = 92	*n* = 32	*n* = 66	*n* = 26	
Caucasian	76 (82.6)	25 (78.1)	56 (84.8)	21 (80.8)	0.76
Asian	9 (9.8)	4 (12.5)	6 (9.1)	3 (11.5)	0.71
Other	6 (6.5)	3 (9.4)	4 (6.1)	2 (7.5)	1.00
Infant characteristcs	*n* = 93	*n* = 32	*n* = 67	*n* = 26	
Sex (Male)	47 (50.5)	18 (56.3)	33 (49.3)	14 (53.9)	0.69
Birth gestation (weeks)	38.7 ± 1.5 ^7^	38.7 ± 1.1	38.5 ± 1.5 ^8^	39.0 ± 1.4 ^4^	0.23
Birth weight (g)	3251 ± 560	3412 ± 578	3242 ± 579	3273 ± 518	0.81
Birth mode	*n* = 91	*n* = 30	*n* = 65	*n* = 26	
Unassisted vaginal	29 (31.9)	8 (26.7)	23 (35.4)	6 (23.1)	0.32
Assisted vaginal	14 (15.4)	3 (10.0)	8 (12.3)	6 (23.1)	0.21
Elective caesarean	28 (30.8)	11 (36.7)	23 (35.4)	5 (19.2)	0.21
Non-elective caesarean	20 (22.0)	8 (26.7)	11 (16.9)	9 (34.6)	0.092

^1^ Data are mean ± standard deviation or *n* (%). ^2^ *p*-value indicates the difference between NMS and LMS groups using Chi-square, Fisher’s exact tests or unpaired Student’s *t*-tests; bold font indicates a significant difference. EP, exclusively pumping mothers with normal milk supply; LMS, low milk supply; MP, milk production; NMS, normal milk supply; PP, predominantly pumping mothers. ^3^ *n* = 71; ^4^ *n* = 25; ^5^ *n* = 52; ^6^ *n* = 19; ^7^ *n* = 91; ^8^ *n* = 66.

**Table 2:** Participants’ perinatal and breastfeeding characteristics.

	PP (*n* = 93)	EP (*n* = 32)	NMS (*n* = 67)	LMS (*n* = 26)	*p*-Value ^2^
Perinatal complications	*n* = 93	*n* = 32	*n* = 67	*n* = 26	
Overall	54 (58.1) ^1^	22 (68.8)	40 (59.7)	14 (53.9)	0.61
Gestational diabetes mellitus	26 (28.0)	11 (34.4)	19 (28.4)	7 (26.9)	1.00
Type 2 diabetes	3 (3.2)	2 (6.3)	3 (4.5)	0 (0.0)	0.56
Preterm birth	9 (9.7)	2 (6.3)	7 (10.6) ^3^	1 (0.4) ^4^	0.44
Gestational hypertension	7 (7.9)	5 (16.7)	7 (10.5)	0 (0.0)	0.18
Foetal growth restriction	6 (6.5)	1 (3.1)	4 (6.0)	2 (7.7)	0.67
Maternal health conditions	*n* = 91	*n* = 30	*n* = 64	*n* = 26	
Overall	50 (54.9)	18 (60.0)	37 (57.8)	13 (50.0)	0.50
Anxiety/depression	25 (28.1) ^5^	7 (23.3)	17 (27.0) ^6^	8 (30.8)	0.80
Polycystic ovary syndrome	17 (19.1) ^5^	8 (26.7)	13 (20.6) ^6^	4 (15.4)	0.77
Fertility issues	10 (11.2) ^5^	4 (13.3)	6 (9.5) ^6^	4 (15.4)	0.47
Infant health conditions	*n* = 93	*n* = 32	*n* = 67	*n* = 26	
Overall	20 (21.5)	11 (34.4)	18 (26.9)	2 (7.7)	0.051
Infant transfer to NICU	5 (5.4)	3 (9.4)	5 (7.5)	0 (0.0)	0.32
Laryngomalacia	2 (2.2)	1 (3.1)	2 (3.0)	0 (0.0)	1.00
Breastfeeding difficulties	*n* = 73	*n* = 25	*n* = 54	*n* = 19	
Attachement difficulties	36 (49.3)	11 (44.0)	26 (48.2)	10 (52.6)	0.74
Nipple pain	30 (41.1)	10 (40.0)	24 (44.4)	6 (31.6)	0.42
Percieved low milk supply	29 (39.7)	5 (20.0)	13 (24.1)	16 (84.2)	**<0.001**
Nipple shield use	23 (31.5)	6 (24.0)	15 (27.8)	8 (42.1)	0.27
Blocked milk ducts	11 (15.1)	4 (16.0)	8 (14.8)	3 (15.8)	1.00
Breast surgery/nipple piercing	8 (8.6)	2 (6.3)	4 (6.0)	4 (15.4)	0.19
Mastitis	6 (8.2)	1 (4.0)	4 (7.4)	2 (10.5)	0.65
Oversupply	5 (6.8)	4 (16.0)	5 (9.3)	0 (0.0)	0.32
Other breastfeeding difficulties	4 (5.5)	2 (8.0)	3 (5.6)	1 (5.3)	1.00
Absence of breastfeeding difficluties	12 (16.4)	5 (20.0)	12 (22.2)	0 (0.0)	**0.029**

^1^ Data are mean ± standard deviation or *n* (%). ^2^ *p*-value indicates the difference between NMS and LMS groups using Chi-square or Fisher’s exact tests; bold font indicates a significant difference. EP, exclusively pumping mothers with normal milk supply; LMS, low milk supply; NMS, normal milk supply; NICU, neonatal intensive care unit; PP, predominantly pumping mothers. ^3^ *n* = 66; ^4^ *n* = 25; ^5^ *n* = 89; ^6^ *n* = 63.

**Table 3:** Maternal conditions—comparison to general population.

Maternal Conditions	PP Frequency	General Population Frequency	Total Sample Size	Chi-Square Statistic Value	*p*-Value ^2^
Mode of birth					
Caesarean section	48 (52.8) ^1^	114,440 (39.0) ^3^	293,526	7.23	**0.007**
Pregnancy complications					
Overall	54 (58.1)	151,084 (46.9) ^4^	322,234	4.65	**0.031**
Gestational diabetes mellitus	26 (28.0)	50,511 (17.9) ^5^	293,528	7.53	**0.006**
Gestational hypertension	7 (7.9)	9205 (3.1) ^5^	293,528	5.89	**0.015**
Health conditions					
Polycystic ovary syndrome	17 (19.1)	31 (12.2) ^6^	343	2.60	0.11
Anxiety/depression	25 (28.1)	1,917,557 (15.7) ^7^	12,213,831	9.53	**0.002**
Breastfeeding difficulties					
Attachment difficulties	36 (49.3)	50 (23.5) ^8^	286	17.27	**<0.001**
Nipple pain	30 (41.1)	169 (36.0) ^9^	542	0.70	0.40
Percieved low milk supply	29 (39.7)	2101 (38.8) ^10^	5487	0.03	0.87
Nipple shield use	23 (31.5)	45 (14.2) ^11^	390	12.35	**<0.001**
Mastitis	6 (8.2)	90 (9.5) ^12^	1019	1.13	0.72
Absence of breastfeeding difficluties	12 (16.4)	497 (90.0) ^13^	625	231.05	**<0.001**

^1^ Data are *n* (%). ^2^ *p*-value indicates the difference between PP and general population groups using Chi-square test for degree of freedom of 1; bold font indicates a significant difference. PP, predominantly pumping mothers. ^3^ *n* = 293,435, Australia 2022 [27]; ^4^ *n* = 322,141, USA 2007–2011 [37]; ^5^ *n* = 242,924, Australia 2022 [29]; ^6^ *n* = 254, Australia 2012–2016 [31]; ^7^ *n* = 12,213,740, Australia 2017–2018 [30]; ^8^ *n* = 213, Brazil 2019 [34]; ^9^ *n* = 469, Australia 2011, nipple pain as a reason for consultation at the breastfeeding centre [32]; ^10^ *n* = 5414, Russian Federation 2022 [36]; ^11^ *n* = 946, Australia 2003, nipple shield use during postnatal hospital stay [33]; ^12^ *n* = 946, USA 1994–1998 [38]; ^13^ *n* = 552, Italy 2018, during third month postpartum [35].

**Table 4:** Twenty-four-hour pumping and breastfeeding characteristics.

Characteristics	EPM (*n* = 32)	NMS (*n* = 67)	LMS (*n* = 26)	*p*-Value ^2^
24 h milk production (g)	1018 ± 274 ^1^	937 ± 263	379 ± 149	**<0.001**
Breastfeeding frequency	0.0 ± 0.0	3.6 ± 4.7	4.1 ± 4.0	0.64
Total amount breastfed (g)	0.0 ± 0.0	110 ± 140	73 ± 83	0.20
Average amount breastfed (g)	0.0 ± 0.0	19.8 ± 29.6	13.7 ± 16.4	0.33
Pumping frequency (per breast)	11.6 ± 2.8	11.1 ± 3.6	9.8 ± 3.4	0.13
Pumping session frequency	6.2 ± 1.8	6.0 ± 2.0	5.2 ± 1.7	0.058
Total expressed (g)	1010 ± 307	822 ± 333	320 ± 137	**<0.001**
EBM feed frequency	7.7 ± 2.6 ^3^	7.3 ± 2.7 ^4^	4.1 ± 2.6	**<0.001**
Total EBM fed (g)	773 ± 184 ^3^	603 ± 235 ^4^	244 ± 161	**<0.001**
Average EBM per feed (g)	108.2 ± 35.0 ^3^	86.7 ± 35.6 ^4^	60.4 ± 35.0	**0.002**
Average amount of breast milk fed (g)	773 ± 184 ^3^	721 ± 172 ^4^	317 ± 182	**<0.001**
Formula feed frequency	0.6 ± 1.5 ^5^	0.6 ± 1.4	4.1 ± 2.8	**<0.001**
Total formula fed (g)	57 ± 123 ^5^	49 ± 108	331 ± 231	**<0.001**
Average formula per feed (g)	26 ± 51 ^5^	24 ± 46 ^6^	73 ± 54	**<0.001**
Total infant milk intake (g) ^7^	843 ± 170	756 ± 178	654 ± 160	**0.014**

^1^ Data are mean ± standard deviation. ^2^ *p*-value indicates significant difference between NMS and LMS groups using unpaired Student’s *t*-test; bold font indicates a significant difference. EBM, expressed breastmilk; EPM, exclusively pumping mothers; LMS, low milk supply; NMS, normal milk supply. ^3^ *n* = 26; ^4^ *n* = 59; ^5^ *n* = 31; ^6^ *n* = 65. ^7^ Includes infant intake of milk via breast, EBM and formula.

**Table 5:** Maternal characteristics and milk removal parameters during pumping session.

**Maternal and Infant Characteristcs**	**PP (*n* = 32)**	**Reference Group (*n* = 60)**	***p*-Value ^2^**
Maternal age at 24 h MP (years)	33.2 ± 4.6 ^1^	32.7 ± 4.4	0.53
Parity (primiparous)	24 (75.0)	38 (63.3)	0.26
Infant birth gestation (weeks)	38.9 ± 1.5	39.0 ± 1.4	0.72
Birth weight (g)	3389 ± 552	3473 ± 527	0.39
Session time (months postpartum)	3.4 ± 1.3	4.2 ± 1.5	**0.003**
24 h milk production (g)	962 ± 338 ^3^	773 ± 233 ^4^	**0.004**
Low milk supply (<600 g/24 h)	3 (11.5) ^3^	10 (17.0) ^4^	0.75
**Milk Removal Parameters**	**PP** (***n* = 54 Sessions**)	**Reference Group** (***n* = 69 Sessions**)	***p*** **-** **Value**
Total milk removed (g)	78 ± 51	93 ± 51	0.090
PAMR (%)	64 ± 34 ^5^	70 ± 18	0.24
Crt pre-expression (%)	3.9 ± 2.4	4.2 ± 2.8	0.56
Crt post-expression (%)	12.6 ± 4.7	13.6 ± 4.6	0.26
DOF pre-expression (%)	0.70 ± 0.23 ^5^	0.74 ± 0.24	0.39
DOF post-expression (%)	0.08 ± 0.13 ^5^	0.12 ± 0.13	0.099

^1^ Data are mean ± standard deviation. ^2^ *p*-value indicates significant difference between PPM and reference groups using unpaired Student’s *t*-test, Chi-square or Fisher’s exact tests where appropriate; bold font indicates a significant difference. Crt, creamatocrit; DOF, degree of fullness of the breast; PAMR, percentage of available milk removed; PP, predominantly pumping mothers. ^3^ *n* = 26; ^4^ *n* = 59; ^5^ *n* = 45.

**Table 6:** Milk ejection and milk flow duration parameters during pumping session.

Parameters	PP (*n* = 52 Sessions)	Reference Group (*n* = 67 Sessions)	*p*-Value ^2^
Milk ejection parameters	*n* = 46	*n* = 64	
Number of ME	5.0 ± 1.4 ^1^	3.7 ± 1.3	**<0.001**
Time to 1st milk flow (min)	0.95 ± 0.88 ^3^	1.01 ± 0.68 ^4^	0.72
Time to 1st ME (min)	1.28 ± 0.87 ^5^	1.47 ± 1.00	0.30
1st ME duration (min)	2.02 ± 0.46	2.04 ± 0.56	0.85
2nd ME duration (min)	1.91 ± 0.40	2.11 ± 0.61	0.050
Peak flow rate 1st ME (g/s)	0.31 ± 0.15	0.51 ± 0.29	**<0.001**
Peak flow rate 2nd ME (g/s)	0.29 ± 0.24	0.42 ± 0.26	**0.011**
Time to peak flow rate 1st ME (g/s)	0.74 ± 0.56	0.74 ± 0.42	0.96
Time to peak flow rate 2nd ME (g/s)	0.56 ± 0.38	0.62 ± 0.28	0.32
Milk during 1st ME (g)	23 ± 13	36 ± 26	**0.002**
Milk during 2nd ME (g)	21 ± 19	29 ± 22	0.051
Milk during 1st ME (%)	33 ± 17	40 ± 19	0.067
Milk during 2nd ME (%)	24 ± 8	30 ± 15	**0.014**
Milk during first two ME (g)	43 ± 27	63 ± 36	**0.002**
Milk during first two ME (%)	58 ± 18	69 ± 17	**0.001**
Flow duration parameters	*n* = 44	*n* = 36	
Total flow duration (min)	12.9 ± 2.6	13.2 ± 3.3	0.69
Active flow duration (min)	11.6 ± 3.2	9.0 ± 3.2 ^6^	**0.002**
Constant flow duration (min)	8.6 ± 4.0 ^3^	7.5 ± 3.7	0.22
Non-flow duration (min)	1.4 ± 1.9	3.6 ± 3.2	**0.009**
Time to stop pumping (min)	11.8 ± 3.0	11.4 ± 3.5	0.65

^1^ Data are mean ± standard deviation. The milk ejection parameters and flow durations’ description is presented in Figure 1. ^2^ *p*-value indicates significant difference between PPM and reference groups using unpaired Student’s *t*-test; bold font indicates a significant difference. ME, milk ejection; PP, predominantly pumping mothers. ^3^ *n* = 45; ^4^ *n* = 36; ^5^ *n* = 52; ^6^ *n* = 67.

**Table 7:** Milk removal efficacy ratios and comfort during pumping session.

Parameters	PP (*n* = 54 Sessions)	Reference Group (*n* = 68 Sessions)	*p*-Value ^2^
Efficacy ratios	*n* = 44	*n* = 32	
Milk removal rate (g/min)	5.2 ± 3.4 ^1,3^	6.3 ± 3.4 ^4^	0.094
Constant flow rate (g/min)	8.2 ± 4.8	10.2 ± 5.0	0.079
Active milk removal (g/min)	7.0 ± 4.1	11.0 ± 6.5 ^5^	**0.001**
Efficacy (g/min)	7.0 ± 4.9 ^6^	7.8 ± 4.0	0.45
Effectiveness (%/min)	5.4 ± 2.2 ^7^	6.9 ± 3.6	**0.044**
Milk after time to stop pumping (g)	2.1 ± 2.4 ^6^	3.7 ± 3.1	**0.011**
Comfort parameters	*n* = 54	*n* = 67	
Expression vacuum (mmHg)	−187 ± 56 ^8^	−221 ± 34 ^9^	**<0.001**
Nipple temperature changes (°C)	−0.6 ± 2.5 ^10^	0.1 ± 2.8 ^11^	0.14
Initial comfort level (1 to 5) ^12^	1.5 ± 0.7	1.6 ± 0.7	0.72
Final comfort level (1 to 5) ^12^	1.5 ± 0.6	1.5 ± 0.6	0.94

^1^ Data are mean ± standard deviation. The efficacy ratios’ description is presented in Figure 1. ^2^ *p*-value indicates significant difference between PPM and reference groups using unpaired Student’s *t*-test; bold font indicates a significant difference. PP, predominantly pumping mothers. ^3^ *n* = 53; ^4^ *n* = 68; ^5^ *n* = 67; ^6^ *n* = 43; ^7^ *n* = 35; ^8^ *n* = 45; ^9^ *n* = 41; ^10^ *n* = 50; ^11^ *n* = 65; ^12^ 1—very comfortable; 2—comfortable; 3—neither comfortable nor uncomfortable; 4—uncomfortable; 5—very uncomfortable.

## Data Availability

Restrictions apply to the availability of some or all data generated or analysed during this study due to ethical reasons. The corresponding author will, on request, detail the restrictions and any conditions under which access to some data may be provided.
